# Synthesis and Biological Evaluation of Dipeptide-Based Stilbene Derivatives Bearing a Biheterocyclic Moiety as Potential Fungicides

**DOI:** 10.3390/molecules27248755

**Published:** 2022-12-09

**Authors:** Yongchuang Zhu, Xingdong Lin, Lan Wen, Daohang He

**Affiliations:** 1 School of Chemical Engineering and Technology, Guangdong Industry Polytechnic, Guangzhou 510300, China; 2 School of Chemistry and Chemical Engineering, South China University of Technology, Guangzhou 510640, China

**Keywords:** stilbene, dipeptide, 1,3,4-oxadiazole, thiophene, antifungal activity, mechanism

## Abstract

The escalating demand for crop production, environmental protection, and food safety warrants the development of new fungicides with greater efficiency, environmental friendliness, and innocuous metabolites to fight against destructive phytopathogens. Herein, we report on the synthesis and antifungal activity of dipeptide-based stilbene derivatives bearing a thiophene-substituted 1,3,4-oxadiazole fragment for the first time. In vitro bioassay indicated that the target compounds had remarkable antifungal potency superior to previously reported counterparts without a dipeptidyl group, of which compound **3c** exhibited the highest activity against *Botrytis cinerea* with EC_50_ values of 106.1 μg/mL. Moreover, the in vivo protective effect of compound **3c** (59.1%) against tomato gray mold was more potent than that of carboxin (42.0%). Preliminary investigations on the mode of action showed that compound **3c** induced marked hyphal malformations and increased the membrane permeability of *B. cinerea* as well as inhibiting mycelial respiration. These promising results suggest that this novel type of molecular framework has great potential to be further developed as alternative fungicides.

## 1. Introduction

Plant fungal infections have remarkably brought about yield reduction and product deterioration as primary plant diseases that have resulted in economic losses in crops [1]. Especially, *Botrytis cinerea* is among the top-ten phytopathogenic fungi given scientific or economic importance, as its causes damage during plant cultivation as well as after harvest [2,3,4]. *B. cinerea*, known as gray mold, is intractable because of its broad host range of more than 220 eudicot plants and manifold attack strategies that include cell wall-degrading enzymes, phytotoxins, and detoxification proteins [5,6]. Several characteristics of chemical fungicides, consisting of low cost, high efficiency, rapid action, and long efficacy duration, remain the mainstay of fungal disease control. Nevertheless, it is the appearance of drug-resistant pathogens [7], and environmental residuals toxic to other organisms [8] resulting from inappropriate or long-term use of available chemicals, that warrant the development of antifungal surrogates with an innovative framework, outstanding bioactivity, and good biocompatibility.

In this context, extensive research efforts have been focused on the exploitation of natural products as an inestimable source of prospective candidates with new modes of action and reasonable degradability. Stilbenes possessing a 1,2-diphenylethylene backbone, a group of plant secondary metabolites, such as well-known resveratrol, are involved in plant defense against multiple biotic and abiotic stresses [9]. Their biological activities [10] and clinical potential [11] have been elaborately documented in the literature. However, although they could inhibit phytopathogenic fungal growth in vitro [12,13], the paucity [10,14], rapid oxidation, and microbial metabolism [15,16] of naturally occurring stilbenes make their external applications on crops hard to achieve, and therefore necessitate synthesis and modification of chemical mimics. Heterocyclic compounds are widely used as components of many biologically active molecules for the optimization of lead compounds in drug development. Thereinto, 1,3,4-oxadiazole substructure prevalent in pesticides and drugs is commonly integrated into the target structures to innovate their biopharmaceutical activities, including antimicrobial [17], anti-inflammatory [18], antitumor [19], and anti-diabetic [20] activities. As another privileged building block, thiophene moiety has drawn the considerable interest of researchers since its derivatives show appreciable diversity in biological effects, such as insecticidal [21], fungicidal [22], and herbicidal [23] effects. Thus, exploration of stilbene backbone modified by these molecular motifs should be expected.

Due to the important role of peptides in living organisms, peptides have been reported as resourceful pharmacological vehicles, and to date, more than 60 peptide therapies have been approved in the United States and other major markets [24,25,26]. Moreover, dipeptide agents, the shortest peptides, are not only easy to prepare and devoid of toxic metabolites, but they are capable of penetrating biological barriers and are more stable when compared to oligopeptides [27]. Dipeptide derivatives, natural and synthetic, have been investigated for applications in diverse aspects [28,29,30,31]. For example, the cyclic Leu-leu dipeptide was isolated and purified from the ethyl acetate extract of a broth of the genus *Gordonia* sp. (WA4-31). Antibacterial experiments showed that it had good inhibitory effects on *Candida albicans*, *Aspergillus niger*, and so on [32]. Khalaf et al. reported Gly-Gly dipeptide derivatives as effective inhibitors against *Bacillus subtilits* and *Candida albicans* [33].

In a previous study, we synthesized a series of 5-(2-thienyl)-1,3,4-oxadiazole-containing stilbene derivatives that displayed promising antifungal activities and could be considered as a potential bioactive scaffold [34]. As a part of our ongoing efforts to develop novel stilbenes of potent fungicidal competence, three different dipeptide fragments (Gly-Gly, Gly-Met, Gly-Leu) were integrated into this stilbene scaffold based on the molecular hybridization principle. The newly synthesized substances were first screened for their in vitro antifungal activities against *B. cinerea*. In addition, their in vivo protective efficacies against tomato gray mold were tested to explore their practical potential in agriculture. The antifungal mechanism of these designed molecules was preliminarily studied in terms of hyphal morphology, membrane permeability, and mycelial respiration.

## 2. Results and Discussion

### 2.1. Chemistry

**Figure 1:**
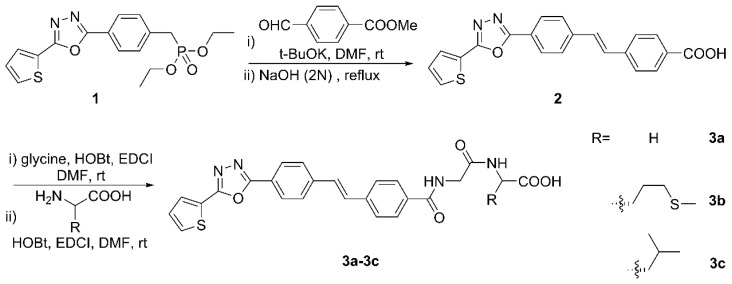
Synthetic route for the target molecules **3a**–**3c**.

Preparation of target compounds **3a**–**3c** was achieved as depicted in Figure 1 starting from the previously described heterocyclic-substituted benzylphosphonate. The stilbene was obtained by the Wittig–Horner reaction of compound **1** with *p*-(Methoxycarbonyl)benzaldehyde, followed by basic hydrolysis to restore the carboxylic acid functionality. The condensation reaction with glycine in the presence of HOBt and EDCI to provide amide was next, which was finally coupled with a certain L-amino acid under the same conditions as one of the former steps for forming the peptide linkage. It is noteworthy that the whole experiment proceeded smoothly without laborious column chromatographic purification, and the target compounds were obtained in good yields. The structures of newly synthesized compounds were confirmed by NMR and HRMS spectral analysis.

### 2.2. In Vitro Antifungal Activities

For the required compounds, the bactericidal activity against two important fungal pathogens was evaluated in vitro. These compounds were preliminarily evaluated at 200 µg/mL, and successfully, the evaluation results (Table 1) revealed that the synthesized molecules showed moderate to good inhibition effects toward the tested fungi. The EC_50_ values for dipeptidyl stilbene derivatives, along with the commercial amide fungicide carboxin, were measured for further exploration of their antifungal potential and are listed in Table 1. It can be clearly seen that compounds **3a**–**3c** were all more potent against *B. cinerea*, with EC_50_ values ranging from 106.1 to 119.6 µg/mL compared to carboxin (EC_50_ = 138.7 µg/mL), out of which **3c** presented the highest level of activity (EC_50_ = 106.1 µg/mL) and was over twice more effective than resveratrol (EC_50_ = 263.1 µg/mL). Despite having less potency against *C. lagenarium*, compounds **3b** and **3c** still exerted fungicidal performance comparable to or better than that of the positive control carboxin. For instance, the EC_50_ value of **3c** was 186.7 µg/mL, lower than that of carboxin (EC_50_ = 201.7 µg/mL). There was an interesting phenomenon in that the final integrated structures displayed growing inhibitory activities with the increasing hydrophobicity of dipeptide substituents due to changes of the external exposed amino acid (Gly < Met < Leu [35]). Furthermore, in contrast with our previous work where the best compound inhibited *B. cinerea* in vitro with EC_50_ = 155.4 μg/mL [36], this test outcome indicated that the introduction of simple dipeptidyl moiety was beneficial to antifungal action.

### 2.3. Effect on Gray Mold of Tomatoes

**Figure 2:**
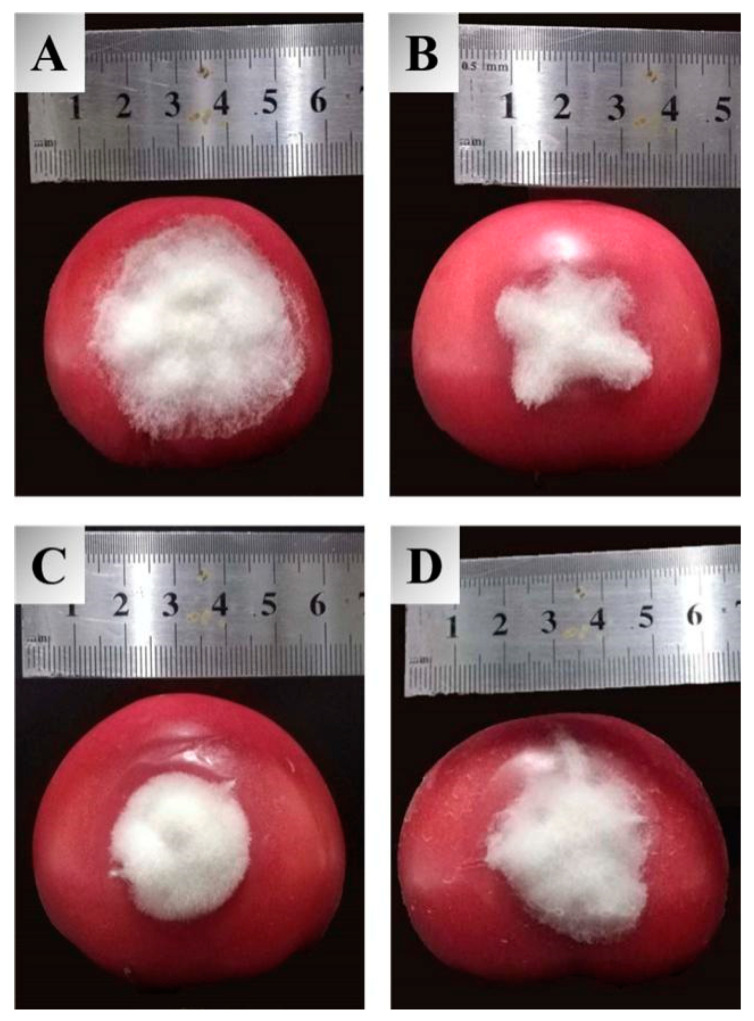
Protective efficacy of compound **3c**, resveratrol, and carboxin against tomato gray mold at 400 µg/mL, (**A**) blank control, (**B**) **3c**, (**C**) resveratrol, (**D**) carboxin.

Inspired by the ameliorated antifungal activities of the target compounds in the in vitro assay, an in vivo experiment for these molecules against tomato gray mold was carried out to examine their practical potential, and the obtained results are summarized in Table 2. The inhibitory effects of synthetic compounds against *B. cinerea* in vivo were identical to those observed against mycelial growth in the Petri dishes. Compounds **3a**–**3c** exhibited excellent protective impacts with the control efficacy of 55.2%, 56.1%, and 59.1%, respectively, at a concentration of 400 µg/mL. Meanwhile, they were all more effective than carboxin (42.0%), among which **3c** can availably prevent the extension of lesions on tomatoes, as is illustrated in Figure 2. Considering the unique chemical structure and efficient bioactivities of dipeptidyl stilbene derivatives containing heterocycles, it is suggested that this type of molecular scaffold could be regarded as prospective agrochemicals for the management of *B. cinerea*.

### 2.4. Optical Microscopy Analysis

**Figure 3:**
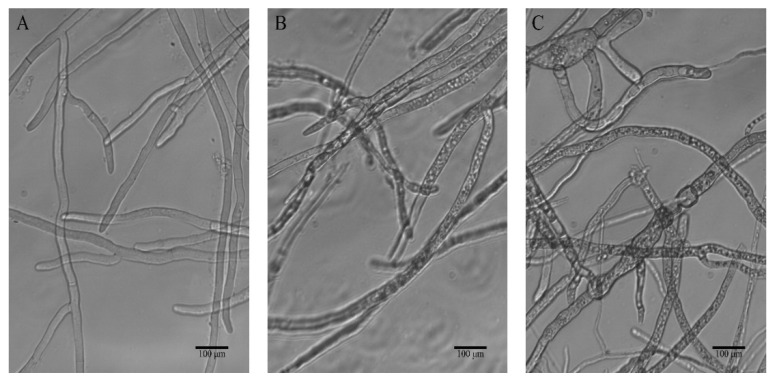
Effect of different concentrations of compound **3c** on the hyphal morphology of *B. cinerea* strains observed by a light microscope (×200), (**A**) blank control, (**B**) 100 µg/mL, (**C**) 200 µg/mL.

The hyphal morphological alterations of *B. cinerea* in response to treatment with **3c** were monitored by light microscopy. In the control sample, the hyphae appeared linear and intact tubular, and homogenous with distinguishable septa, while as shown in Figure 3B, the fungus subjected to the action of compound **3c** at 100 µg/mL presented evident changes, of which hyphal vesiculation was particularly visible. The imprints on the mycelia were more significant after exposure to 200 µg/mL **3c**, and microscopic examination showed irregularly tortuous hyphae without a relatively uniform diameter, swollen or elongated. These observed mycelial malformations implied that destroying the structure of the cell wall and membrane system might be one of the antifungal mechanisms of compound **3c** against *B. cinerea*.

### 2.5. Effect on Cell Membrane Permeability

**Figure 4:**
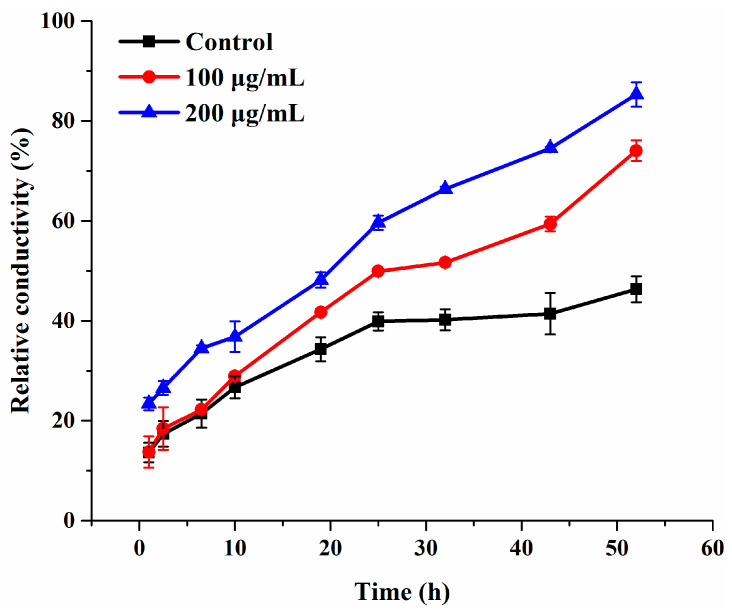
Electrolyte leakage from *B. cinerea* suspensions treated with compound **3c**. Each treatment was carried out in triplicate.

Cell membrane plays a pivotal role in maintaining cellular basic metabolism and defending the cell against exogenous disturbance [36]. Electrolyte leakage is generally taken as an indicator of cell membrane permeability. Hence, in order to confirm whether compound **3c** affects the membrane permeability of *B. cinerea*, the electrical conductivity of suspensions of intact mycelia was measured. Compared to the control group, the relative permeability in the **3c**-treated groups was higher and continually elevated during the entire time of treatment, even 25 h later (Figure 4). Besides, the extent of the damage induced by **3c** to the mycelial cell membrane system increased in a concentration-dependent manner, which was distinctly revealed by significant effects at 200 µg/mL within 10 h. These findings proved that the exposure to compound **3c** permeabilized the membrane, led to electrolyte leakage and thereby augmented the conductivity of the solution.

### 2.6. Effect on Mycelial Respiration

**Figure 5:**
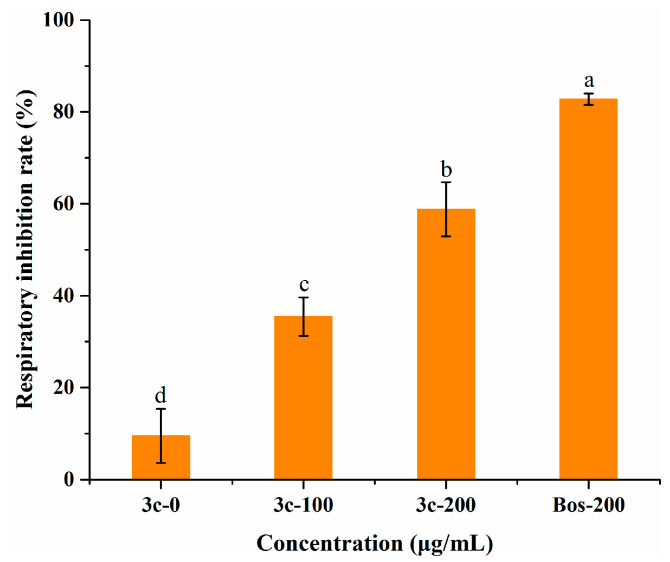
Evaluation of the respiratory inhibition rate on *B. cinerea* mycelia exposed to compound **3c** and the commercial respiration inhibitor boscalid (BOS). Different letters among treatments mean statistically significant differences using Tukey’s test (*p* < 0.05).

According to related reports, pterostilbene can interact with the mitochondrial membrane of cells and inhibit cell respiration [37]. To further explore the antifungal mechanism, we tested the mycelial oxygen consumption rate of *B. cinerea* when treated with compound **3c**, utilizing a respiration inhibitor, boscalid, as a reference. Notwithstanding that the respiration inhibitory activity (58.8 ± 5.9%) was poorer than that of boscalid (82.8 ± 1.2%) at an equal dose (Figure 5), treatment with **3c** had a statistically significant impact on mycelial respiration, which suggested that this compound might also function in the same way as boscalid and partly suppress the mycelium growth of *B. cinerea* by inhibiting the mitochondrial respiratory chain. Likewise, the low level of compound **3c** resulted in less prominent inhibition of oxygen consumption. Combined with the results of the membrane permeability assay, it was inferred that **3c** exerted antifungal activity against *B. cinerea* through multiple pathways, favorable to retarding the development of fungal resistance.

To sum up, we designed and synthesized structurally tunable dipeptide-based stilbene derivatives bearing thiophene and 1,3,4-oxidazole moieties by innovatively introducing easy-to-prepare and diverse dipeptides. The in vitro bioassay indicated that all the compounds possessed better fungicidal activities against tested fungal strains than that of resveratrol. Furthermore, in vivo experiments demonstrated their effectiveness for the control of tomato gray mold caused by *B. cinerea*. It was also observed that abnormal hyphal morphology, increased membrane permeability, and the repressed mycelial respiration of *B. cinerea* took place after interaction with compound **3c**. Given their high efficacies and versatile effects on gray mold, dipeptidyl stilbenes are of promising development value for seeking novel agricultural fungicides.

## 3. Materials and Methods

### 3.1. Chemicals and Instrumentation

All chemicals, reagents, and solvents were obtained commercially and used without further purification. Melting points were determined employing a BUCHI Melting Point M-565. ^1^H, and ^13^C NMR spectra were recorded in deuterated dimethyl sulfoxide (DMSO-*d*_6_) on a Bruker AVANCE 400 (400 MHz for ^1^H and 100 MHz for ^13^C) spectrometer (Switzerland) using TMS as an internal standard. Chemical shifts (*δ*) were expressed in parts per million (ppm) and coupling constants (*J*) were given in hertz (Hz). The following abbreviations were used to explain the multiplicities: s = singlet; d = doublet; t = triplet; q = quartet; m = multiplet. Mass spectra were registered on a high-resolution electrospray ionization mass spectrometer (maXis impact, Bruker, Germany).

### 3.2. In Vitro Antifungal Test

The in vitro fungicidal activity of the target compounds was demonstrated by examining the inhibition of the mycelial radial growth of representative phytopathogenic fungus, *B. cinerea*. These compounds were initially diluted to the testing concentration of 200 µg/mL with PDA medium at 50–60 °C and transferred into sterilized Petri dishes. After solidification, 5 mm-diameter mycelial disks taken from the peripheral part of a colony of each active fungus were placed at the center of the dishes aseptically. The plates were then incubated at 25 °C for 3 days. DMSO served as the negative control whereas carboxin served as the positive control. Each experiment was carried out in triplicate. The inhibitory effect of the test compounds on both fungi was calculated by the formula I=(1 − a/b) × 100%, where *I* represented the inhibition rate, and *a* and *b* were the mean colony diameters in the untreated and treated Petri dishes, respectively. Additionally, the corresponding inhibition rates of all test compounds at a series of concentrations (400, 200, 100, 50, 25, 12.5, 6.25 µg/mL) were measured under the same conditions as described above to compute medium effective concentration (EC_50_) values with SPSS 17.0 software.

### 3.3. In Vivo Assay against Tomato Gray Mold

In vivo assay was conducted on tomatoes (*Lycopersicum esculentum*) artificially inoculated with *B. cinerea*. Fruits were selected as experimental material based on uniformity and absence of physical injuries or infections, then surfaced-disinfected with 75% ethanol, rinsed with tap water, and air-dried before treatment. Test solutions (400 µg/mL) of synthesized compounds, prepared by dissolution in DMSO and dilution with distilled water comprising 0.1% Tween 80, were sprayed evenly on fruits and allowed to dry at room temperature. Commercial carboxin and an equivalent amount of DMSO were used as controls. The fruits were wounded with a sterile inoculation needle at the equatorial region and inoculated with the pathogen afterward. Each treatment consisted of three replicates. Treated fruits were stored at 25 °C and high relative humidity (90–95%) for one week. The efficacy of test compounds was expressed by the percentage of reduction in lesion diameter that was determined using the cross method.

### 3.4. Optical Microscopy of Hyphal Morphology of B. cinerea

Three-day-old *B. cinerea* mycelia were cultured in 50 mL potato dextrose broth (PDB) containing different concentrations of **3c** (100 µg/mL and 200 µg/mL). The blank control had equal content (0.5%) of DMSO. After incubation at 25 °C for 22.5 h, the mycelia were collected, washed with 0.2 M phosphate-buffered saline (PBS) at pH 7.2, resuspended in PBS (0.2 M, pH 7.2), and observed using a light microscope at ×200 magnification.

### 3.5. Assessment of Cell Membrane Permeability

The change in the membrane permeability of *B. cinerea* was examined by measuring relative conductivity with a DDS-307 conductivity meter (Shanghai INESA Scientific Instrument Co. Ltd., Shanghai, China). The mycelia of 3-day-old *B. cinerea* were collected from PDB medium and washed with sterile distilled water, then treated with 100 or 200 µg/mL compound **3c**. 0.01% DMF at the same dosage as the solutions mentioned above, and served as the blank control. Thereafter, the electrical conductivity of the mycelia suspension was determined at 0 (*L*_0_), 1, 2.5, 6.5, 10, 19, 25, 32, 43, and 52 h (*L*_1_) with the final conductivity (*L*_2_) of mycelia suspension being after it was boiled and cooled. The equation for the relative permeability was ${p=[(L}_{1}{-L}_{0}{)/(L}_{2}{-L}_{0})]\;\times \;100\%$.

### 3.6. Determination of Oxygen Consumption

The influence of compound **3c** on the mycelial respiration of *B. cinerea* was evaluated as stated by Yan et al [38]. Mycelial blocks from a one-week-old culture of *B. cinerea* were placed in 50 mL PDB and incubated at 25 °C for 3 days with 200 rpm shaking. After being harvested and rinsed, mycelia were suspended in 0.1 M PBS (pH 7.2, 50 mg fresh weight of mycelia mL^−1^) amended with 2% glucose, comprising **3c** (100 or 200 µg/mL) or boscalid (200 µg/mL, positive control) and 0.01% DMF (blank control). Each treatment was repeated three times. A JPB-607A dissolved oxygen meter (Shanghai INESA Scientific Instrument Co. Ltd., Shanghai, China) was employed to determine mycelial oxygen consumption. The inhibition rate of respiration (IR) was calculated by the following formula $I_{R}=(1-{\;R}_{1}{/\;R}_{0})\;\times \;100\%$, where *R*_1_ and *R*_0_ were the ratios of mycelial oxygen uptake with or without treatment, respectively. Statistical analysis of the results was performed using SPSS 17.0.

### 3.7. General Method for the Synthesis of Carboxylic Acid 2

Phosphonate **1** (1.89 g, 5 mmol) prepared according to the procedure reported (see Supplementary Materials) was dissolved in DMF (30 mL) and added to a 100 mL flask containing *p*-(Methoxycarbonyl)benzaldehyde (0.82 g, 5 mmol); then, an ethanol solution of potassium *tert*-butoxide (15 mL, 6 mmol) was introduced and the mixture was stirred for 6 h. The formed precipitate was filtered, washed with water, and dried to obtain intermediate ester.

The hydrolyzation of ester was carried out with 2 N NaOH aqueous solution under reflux (6 h). After cooling to room temperature, the solution was acidified to pH 3–4 with 1 M HCl, followed by filtration to obtain a crude product that was purified by recrystallization from DMSO/EtOH to give 1.31 g of **2**.

### 3.8. General Method for the Synthesis of the Target Compounds

A two-step synthetic procedure was applied to obtain the target compounds. Acid **2** (1.20 g, 3.20 mmol) was stirred for 2 h in DMF (30 mL) at ambient temperature with 1-hydroxybenzotriazole (HOBt, 0.43 g, 3.20 mmol) and 1-(3-dimethylaminopropyl)-3-ethylcarbodiimide hydrochloride (EDCI, 0.61 g, 3.20 mmol). Glycine (0.24 g, 3.20 mmol) was then added and the reaction system was left to stir for 7 h. Then, 50 mL of water was added and the resultant yellow solid was removed by filtration and washed successively with 0.5 M HCl and methanol to remove impurities.

The coupling of the above resultant with glycine, L-methionine and L-leucine, respectively, was performed in a similar manner to its own synthetic protocols to afford target compounds **3a**–**3c** that were recrystallized from DMSO/EtOH.

#### 3.8.1. (E)-4-(4-(5-(Thiophen-2-yl)-1,3,4-oxadiazol-2-yl)styryl)benzoic acid (**2**)

^1^H NMR (400 MHz, DMSO-d6) δ 12.91 (s, COOH, 1H), 8.07 (d, *J* = 8.0 Hz, Th-H, 2H), 7.96 (d, *J* = 7.5 Hz, Ph-H, 4H), 7.85 (d, *J* = 8.1 Hz, Ph-H, 2H), 7.75 (d, *J* = 8.0 Hz, Ph-H, 2H), 7.48 (s, CH = CH, 2H), 7.32 (t, *J* = 4.3 Hz, Th-H, 1H); ^13^C NMR (101 MHz, DMSO-d6) δ 167.46, 163.76, 160.78, 141.38,140.65, 132.12, 131.00, 130.45, 130.29,130.24, 130.18, 129.20, 128.05, 127.49, 127.27, 124.76, 122.67; HRMS (ESI), m/z calcd for C_21_H_15_N_2_O_3_S [M + H] + 375.0798; found, 375.0793.

#### 3.8.2. (E)-(4-(4-(5-(Thiophen-2-yl)-1,3,4-oxadiazol-2-yl)styryl)benzoyl)glycylglycine (**3a**)

A yellow solid, yield 54%, mp 217–218 °C; ^1^H NMR (400 MHz, DMSO-*d*_6_) *δ* 12.56 (s, 1H), 8.86 (dt, *J* = 17.9, 5.7 Hz, 1H), 8.24 (t, *J* = 5.9 Hz, 1H), 8.12 (d, *J* = 8.0 Hz, 2H), 7.99 (t, *J* = 4.0 Hz, 2H), 7.94 (d, *J* = 8.1 Hz, 2H), 7.89 (d, *J* = 8.1 Hz, 2H), 7.78 (d, *J* = 8.1 Hz, 2H), 7.52 (s, 2H), 7.35 (t, *J* = 4.4 Hz, 1H), 3.94 (d, *J* = 5.8 Hz, 2H), 3.79 (d, *J* = 5.8 Hz, 2H); ^13^C NMR (101 MHz, DMSO-*d*_6_) *δ* 171.64, 169.83, 166.47, 163.80, 160.80, 140.84, 139.97, 133.71, 132.19, 131.06, 130.47, 129.54, 129.26, 128.39, 127.99, 127.54, 127.06, 124.77, 122.55, 42.93, 41.18; HRMS (ESI) [M + Na]^+^ calcd for C_25_H_20_N_4_O_5_S: 511.1047, found: 511.1040.

#### 3.8.3. (E)-(4-(4-(5-(Thiophen-2-yl)-1,3,4-oxadiazol-2-yl)styryl)benzoyl)glycyl-L-methionine (**3b**)

A yellow solid, yield 50%, mp 196–197 °C; ^1^H NMR (400 MHz, DMSO-*d*_6_) *δ* 12.61 (s, 1H), 8.76 (t, *J* = 6.0 Hz, 1H), 8.24 (d, *J* = 7.9 Hz, 1H), 8.11 (d, *J* = 8.0 Hz, 2H), 7.98 (t, *J* = 4.0 Hz, 2H), 7.93 (dd, *J* = 8.3, 3.4 Hz, 2H), 7.88 (d, *J* = 8.1 Hz, 2H), 7.77 (d, *J* = 8.1 Hz, 2H), 7.51 (s, 2H), 7.34 (t, *J* = 4.4 Hz, 1H), 4.38 (td, *J* = 8.5, 4.5 Hz, 1H), 4.03–3.89 (m, 2H), 2.55 (s, 4H), 2.05 (s, 3H); ^13^C NMR (101 MHz, DMSO-*d*_6_) *δ* 173.67, 169.55, 166.48, 163.80, 160.80, 140.84, 139.95, 133.75, 132.18, 131.06, 130.47, 129.54, 129.26, 128.33, 127.99, 127.54, 127.08, 124.76, 122.55, 51.41, 40.90, 31.30, 30.11, 15.05; HRMS (ESI) [M + H]^+^ calcd for C_28_H_27_N_4_O_5_S_2_: 563.1378, found: 563.1418.

#### 3.8.4. (E)-(4-(4-(5-(Thiophen-2-yl)-1,3,4-oxadiazol-2-yl)styryl)benzoyl)glycyl-L-leucine (**3c**)

A yellow solid, yield 56%, mp 215–216 °C; ^1^H NMR (400 MHz, DMSO-*d*_6_) *δ* 12.68 (s, 1H), 8.85–8.71 (m, 1H), 8.18 (d, *J* = 8.1 Hz, 1H), 8.12 (s, 2H), 7.99 (d, *J* = 5.0 Hz, 2H), 7.93 (d, *J* = 7.1 Hz, 2H), 7.88 (d, *J* = 8.0 Hz, 2H), 7.77 (d, *J* = 5.8 Hz, 2H), 7.51 (s, 2H), 7.34 (d, *J* = 4.4 Hz, 1H), 4.29 (td, *J* = 8.6, 5.9 Hz, 1H), 3.96 (t, *J* = 4.5 Hz, 2H), 1.67 (q, *J* = 6.8 Hz, 1H), 1.54 (dq, *J* = 12.6, 7.9, 6.4 Hz, 2H), 0.89 (dd, *J* = 16.2, 6.5 Hz, 6H); ^13^C NMR (101 MHz, DMSO-*d*_6_) *δ* 174.49, 171.82, 169.38, 166.46, 163.80, 160.79, 140.84, 139.93, 133.79, 133.54, 132.18, 131.06, 130.44, 129.25, 128.32, 128.24, 127.99, 127.54, 127.15, 127.08, 124.77, 122.55, 24.76, 23.33, 21.91; HRMS (ESI) [M + H]^+^ calcd for C_29_H_29_N_4_O_5_S: 545.1814, found: 545.1856.

## Figures and Tables

**Table 1:** In vitro antifungal activities of the target compounds against *B. cinerea*.

Compound	Inhibition Rate (%) at 200 µg/mL *^a^*	EC_50_ (µg/mL)
**3a**	73.0 ± 1.2 b	119.6
**3b**	74.8 ± 1.7 b	116.3
**3c**	79.6 ± 2.6 a	106.1
Carboxin	77.9 ± 1.6 ab	138.7
Resveratrol *^b^*	44.5 ± 1.2 c	263.1

*^a^* Means followed by different letters within each column are significantly different (Tukey’s test, *p* < 0.05). *^b^* Data from our previous report under the same test conditions [36].

**Table 2:** Protective activities of compounds **3a**–**3c** against tomato gray mold at 400 µg/mL under greenhouse conditions.

Compound	Leision Length (mm) *^a^*	Control Efficacy (%)
**3a**	24.7 ± 2.6 c	55.2%
**3b**	24.3 ± 1.3 c	56.1%
**3c**	23.0 ± 1.5 c	59.1%
Carboxin	30.5 ± 1.4 b	42.0%
Resveratrol	25.0 ± 2.2 c	54.5%
CK *^b^*	49.0 ± 1.0 a	

*^a^* Results are means of three independent replicates ± SD; means with different letters are significantly different (Tukey’s test, *p* < 0.05). *^b^* Blank Control.

## Data Availability

Data is contained within the article.

## Supplementary Materials

The following supporting information can be downloaded at: https://www.mdpi.com/article/10.3390/molecules27248755/s1, Synthetic procedure and characterization data for intermediate 1, citation of references [34,39]

## Sample Availability

Samples of the compounds are not available from the authors.
