# Supplementary material for: Synthesis and Biological Evaluation of Dipeptide-Based Stilbene Derivatives Bearing a Biheterocyclic Moiety as Potential Fungicides
Source: Molecules. 2022 Dec 9;27(24):8755. doi: 10.3390/molecules27248755 (PMC9784524; doi:10.3390/molecules27248755)
Supplement: Supplementary file 1 [file molecules-27-08755-s001.zip › molecules-2019698-supplementary.pdf]

molecules

Article

Synthesis and Biological Evaluation of Dipeptide-Based Stilbene Derivatives Bearing a Biheterocyclic Moiety as Potential Fungicides

Content

Synthetic procedure and characterization data for intermediate 1 2

References 2

Synthetic procedure and characterization data for intermediate 1.

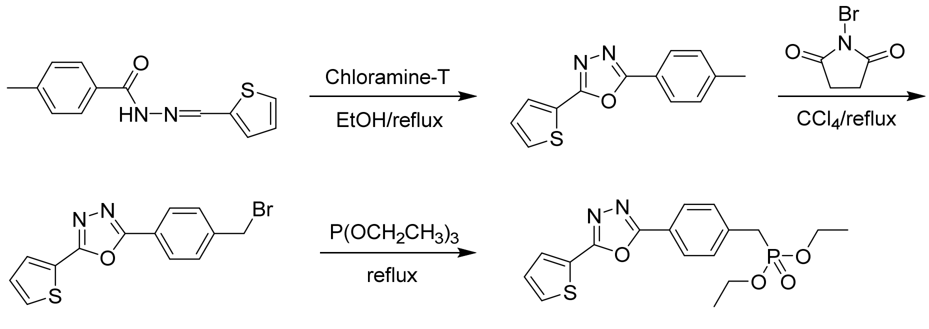

1

*Synthesis of intermediate **1***. A mixture of benzyl bromide [1,2] (3.21 g, 10 mmol) and triethyl phosphite (30 mL) was heated under reflux for 5 h. The excess triethyl phosphite was evaporated under reduced pressure, and then filtered after addition of hexane. The residue was recrystallized from ethanol to afford compound **1**.

Diethyl (4-(5-(thiophen-2-yl)-1,3,4-oxadiazol-2-yl)benzyl)phosphonate (1). A light yellow solid, yield 72.3%; ^{1}H NMR (400 MHz, $CDCl_3$) δ 8.07 (d, *J* = 8.0 Hz, 2H), 7.84 (dd, *J* = 3.7, 1.2 Hz, 1H), 7.58 (dd, *J* = 5.0, 1.2 Hz, 1H), 7.48 (dd, *J* = 8.3, 2.5 Hz, 2H), 7.20 (dd, *J* = 5.0, 3.7 Hz, 1H), 4.05 (qd, *J* = 8.4, 7.7, 6.5 Hz, 4H), 3.23 (d, *J* = 22.1 Hz, 2H), 1.27 (t, *J* = 7.1 Hz, 6H); ^{13}C NMR (101 MHz, $CDCl_3$) δ 160.83, 136.07, 135.98, 130.55, 130.49, 130.17, 129.77, 128.19, 127.12, 127.08, 125.21, 62.37, 62.30, 34.71, 33.34, 16.43, 16.37.

References

1. Wen, L.; Jian, W. L.; Shang, J. B.; He, D. H. Synthesis and antifungal activities of novel thiophene-based stilbene derivatives bearing an 1,3,4-oxadiazole unit. *Pest Manag Sci* **2019**, 75, 1123-1130.
2. Jian, W. L.; He, D. H.; Xi, P. G..; Li X. W. Synthesis and biological evaluation of novel fluorine-containing stilbene derivatives as fungicidal agents against phytopathogenic fungi. *J. Agric. Food. Chem.* **2015**, 63, 9963-9969.
